# Investigation of the Defective Growth Pattern and Multidrug Resistance in a Clinical Isolate of Candida glabrata Using Whole-Genome Sequencing and Computational Biology Applications

**DOI:** 10.1128/spectrum.00776-22

**Published:** 2022-07-18

**Authors:** Osman Merdan, Ayşe Sena Şişman, Seçil Ak Aksoy, Samet Kızıl, Nazmiye Ülkü Tüzemen, Emel Yılmaz, Beyza Ener

**Affiliations:** a Department of Medical Microbiology, Faculty of Medicine, Bursa Uludağ University, Bursa, Turkey; b Department of Clinical Microbiology and Infectious Diseases, Faculty of Medicine, Bursa Uludağ University, Bursa, Turkey; c İnegöl Vocational School, Bursa Uludağ University, Bursa, Turkey; University of Guelph

**Keywords:** amphotericin resistance, azole resistance, *Candida glabrata*, cholesterol dependent, ERG1, whole-genome sequencing

## Abstract

Candida glabrata is increasingly isolated from blood cultures, and multidrug-resistant isolates have important implications for therapy. This study describes a cholesterol-dependent clinical C. glabrata isolate (ML72254) that did not grow without blood (containing cholesterol) on routine mycological media and that showed azole and amphotericin B (AmB) resistance. Matrix-assisted laser desorption ionization–time of flight (MALDI-TOF) and whole-genome sequencing (WGS) were used for species identification. A modified Etest method (Mueller-Hinton agar supplemented with 5% sheep blood) was used for antifungal susceptibility testing. WGS data were processed via the Galaxy platform, and the genomic variations of ML72254 were retrieved. A computational biology workflow utilizing web-based applications (PROVEAN, AlphaFold Colab, and Missense3D) was constructed to predict possible deleterious effects of these missense variations on protein functions. The predictive ability of this workflow was tested with previously reported missense variations in ergosterol synthesis genes of C. glabrata. ML72254 was identified as C. glabrata *sensu stricto* with MALDI-TOF, and WGS confirmed this identification. The MICs of fluconazole, voriconazole, and amphotericin B were >256, >32, and >32 μg/mL, respectively. A novel frameshift mutation in the *ERG1* gene (Pro314fs) and many missense variations were detected in the ergosterol synthesis genes. None of the missense variations in the ML72254 ergosterol synthesis genes were deleterious, and the Pro314fs mutation was identified as the causative molecular change for a cholesterol-dependent and multidrug-resistant phenotype. This study verified that web-based computational biology solutions can be powerful tools for examining the possible impacts of missense mutations in C. glabrata.

**IMPORTANCE** In this study, a cholesterol-dependent C. glabrata clinical isolate that confers azole and AmB resistance was investigated using artificial intelligence (AI) technologies and cloud computing applications. This is the first of the known cholesterol-dependent C. glabrata isolate to be found in Turkey. Cholesterol-dependent C. glabrata isolates are rarely isolated in clinical samples; they can easily be overlooked during routine laboratory procedures. Microbiologists therefore need to be alert when discrepancies occur between microscopic examination and growth on routine media. In addition, because these isolates confer antifungal resistance, patient management requires extra care.

## INTRODUCTION

*Candida* species are opportunist pathogens responsible for bloodstream infections in humans. C. glabrata is the second or third most frequently isolated *Candida* species from blood cultures in developed countries, and it ranks third after C. albicans and C. parapsilosis in Turkey. It is responsible for roughly 15% of all candidemia cases (1, 2).

Several studies have shown that delays in the treatment of candidemia are associated with increased mortality (3, 4). Current guidelines recommend echinocandins, followed by fluconazole and amphotericin B (AmB), as first-choice drugs for candidemia (5, 6). Improvements in antifungal susceptibility testing methods for detection of the resistance of *Candida* isolates have also provided better regional and worldwide epidemiological data (7, 8). Shifts in *Candida* epidemiology to more resistant species, and especially multidrug-resistant C. glabrata, have created a challenge for antifungal treatment of candidemia in critically ill patients (9). The increased use of azoles and echinocandins is also believed to have imposed selection pressure on C. glabrata, resulting in resistant strains (10). The ARTEMIS Antifungal Surveillance Program reported an increase in fluconazole-resistant C. glabrata isolates from 9% to 14% during the 2001 to 2007 period compared to the 1992 to 2001 period. Echinocandin resistance has shown a similar tendency to increase, with a range of 3 to 12% (11, 12).

**FIG 1:**
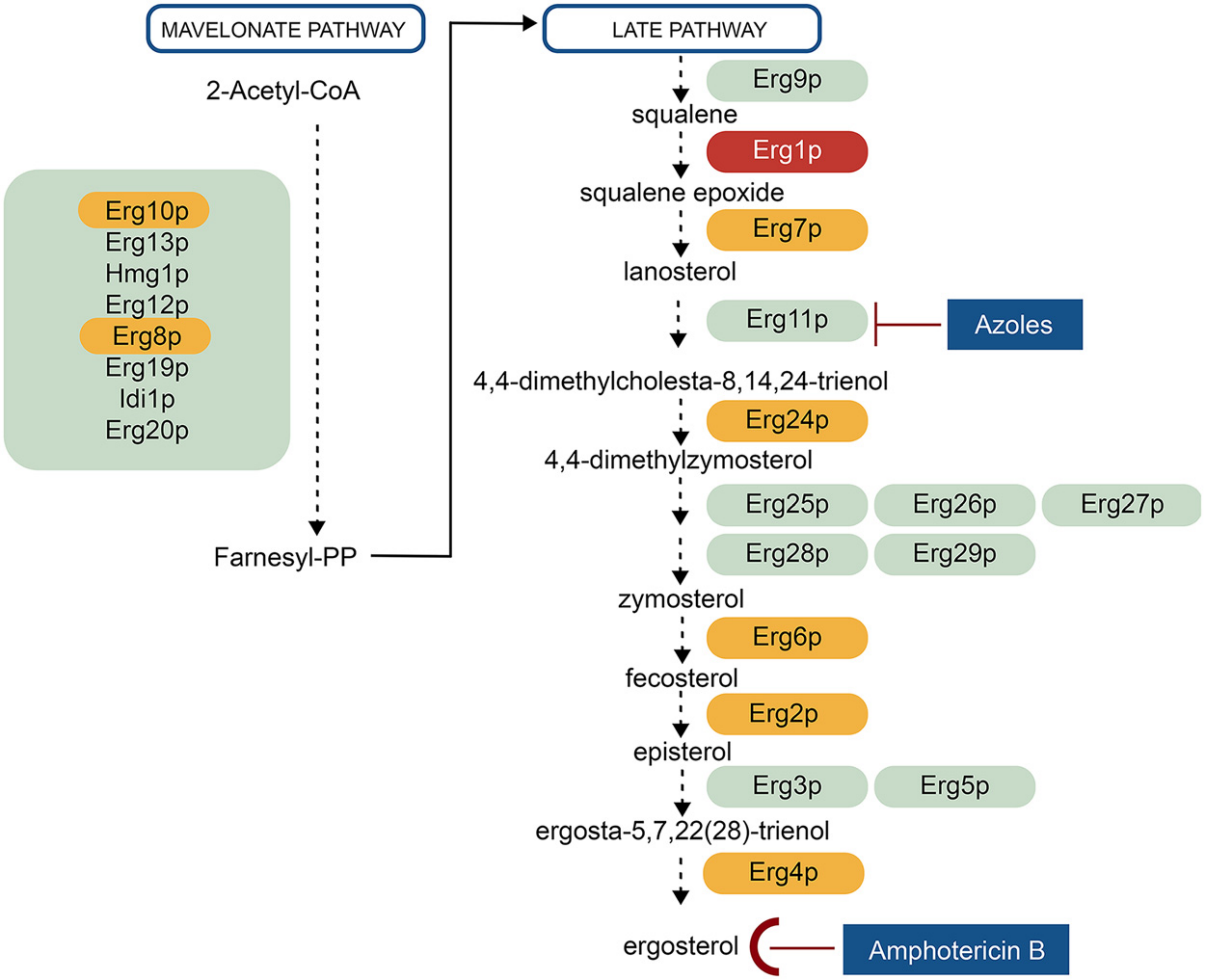
Ergosterol biosynthetic pathway. The ML72254 strain proteins shown in green are with no nonsynonymous variations; shown in orange are those with nonsynonymous variations that do not cause loss of function; shown in red is mutant protein causing functional loss.

For echinocandin- and azole-resistant C. glabrata, AmB is the drug of choice for treatment (13). Although amphotericin B resistance is not common in C. glabrata strains, increased resistance rates are observed in some geographical regions and in biofilm-forming strains (14–17). AmB intercalates directly with membrane ergosterol, the principal sterol in fungal membranes, to form channels that leak monovalent ions and extramembranous aggregates that extract ergosterol from the phospholipid bilayer, causing yeast cell death (18). Ergosterol is responsible for cell membrane integrity and the proper functioning of membrane proteins, making it a perfect target for antifungal drugs. Azole drugs disrupt ergosterol synthesis while inhibiting the function of lanosterol 14 α-demethylase, the product of the *ERG11* gene (19, 20) (Fig. 1).

Deleterious mutations of essential genes within the late pathway of ergosterol synthesis can completely eliminate ergosterol production in C. glabrata and cause growth defects when cultured on sterol-poor medium (e.g., Sabouraud’s dextrose agar [SDA]). However, C. glabrata imports sterol under both aerobic and anaerobic conditions as shown by Zavrel et al. (21). Sterol import under low oxygen levels is dependent on the transcription factor Upc2, but a block in ergosterol synthesis and serum supplementation (containing cholesterol) also induces aerobic sterol uptake in C. glabrata, and growth can be restored (21). C. glabrata isolates with these phenotypic characteristics are referred to as cholesterol-dependent (22–24), and they show distinct growth characteristics and antifungal susceptibility patterns due to different mutations in the late pathway of ergosterol synthesis (25).

The aim of this study was to investigate a clinical isolate of cholesterol-dependent C. glabrata (ML72254) that did not grow on routine mycological media and that showed both azole and AmB resistance. A second goal was to explore the molecular changes in the ML72254 genome using web-based computational biology applications to characterize the mutation that led to defective growth and drug resistance.

## RESULTS

### Species identification and growth characteristics.

**FIG 2:**
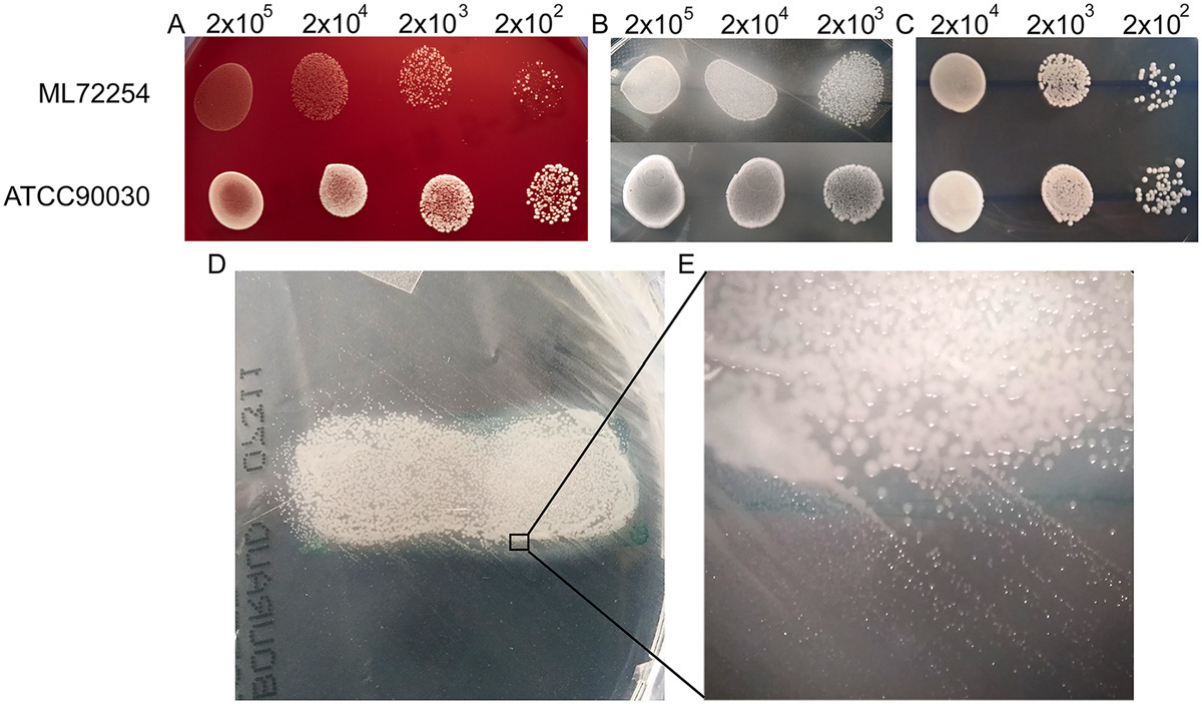
(A to C) Growth of the Candida glabrata ML72254 and ATCC 90030 strains on sheep blood agar (SBA) (A), Sabouraud’s dextrose agar-human serum (SDA-HS) (B), and Sabouraud’s dextrose agar-cholesterol supplemented (60 μg/mL) (SDA-CS) (C) plates after a 72-h incubation under standard atmospheric conditions. The SBA agar plate showed a few small ML72254 colonies after inoculation at 2 × 10^2^ cells/mL. ATCC 90030 showed more abundant growth than ML72254 on SBA and SDA-HS plates (A and B). ML72254 grew on the SDA-CS medium and formed colonies similar in size those of to ATCC 90030 (C). ML72254 grew only in the area with added human serum on the SDA plate (D). (E) A magnified version of the image in panel D indicated that colony size decreased as the distance to the serum-containing area increased.

The ML72254 strain was identified as C. glabrata *sensu stricto* with matrix-assisted laser desorption ionization–time of flight (MALDI-TOF), and whole-genome sequencing (WGS) confirmed this identification. After a 72 h of incubation on the SBA (Columbia agar with 5% sheep blood) plate, the ML72254 isolate formed 0.2- to 0.5-mm-diameter flat gray colonies, as opposed to the white 1- to 3-mm-diameter colonies formed by the C. glabrata ATCC 90030 reference strain (Fig. 2). Cholesterol supplementation of SDA plates (60 μg/mL) supported the growth of ML72254, and after 72 h of incubation, C. glabrata ATCC 90030 and ML72254 colonies were similar in size on SDA plates supplemented with 60 μg/mL cholesterol (SDA-CS plates) (Fig. 2). Growth was detected only in the area containing human serum on SDA plates. As shown in Fig. 2, colony sizes decreased in the peripheral regions, indicating the need for sterols.

**FIG 3:**
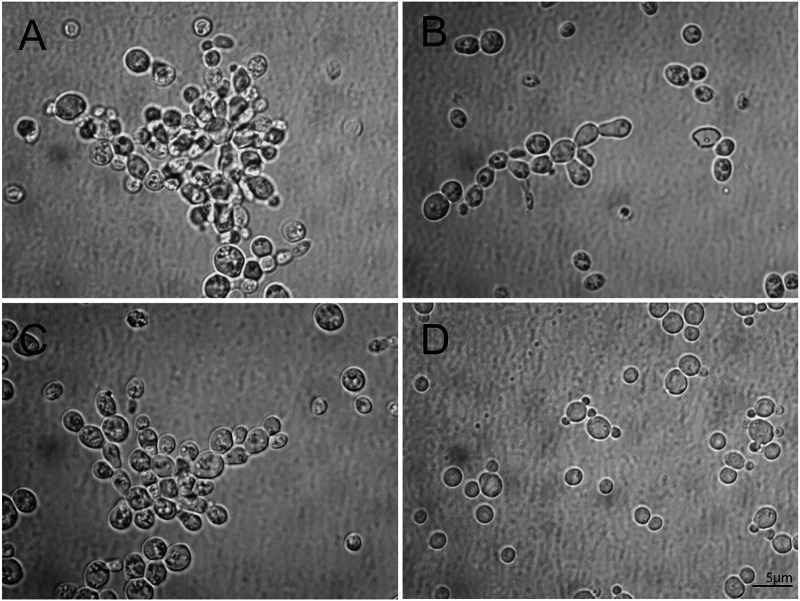
Pseudohyphal-like growth of the Candida glabrata ML72254 strain was observed by direct microscopy after incubation on a Sabouraud dextrose agar-human serum (SDA-HS) plate under standard atmospheric conditions. (A to C) Cells were clumped together and showed branching patterns. (D) C. glabrata reference strain ATCC 90030 grown on SDA-HS showed solitary and budding cells.

Microscopy examination of the cells of the ML72254 strain after incubation on SDA plates supplemented with human serum (SDA-HS) revealed pseudohypha-like growth. As illustrated in Fig. 3, the cells of the isolate were arranged in bundles and in branching patterns.

### Antifungal susceptibility testing results.

The *in vitro* antifungal susceptibilities of C. glabrata ML72254 and ATCC 90030 are shown in Table 1. The ML72254 strain was susceptible to echinocandins but resistant to AmB and azoles. The MICs of C. glabrata ATCC 90030 and the other control strains were within the susceptible range.

**TABLE 1:** *In vitro* susceptibility of the Candida glabrata ML72254 and ATCC 90030 strains

Antibiotic	ECOFF^*a*^ (μg/mL)	MIC (μg/mL)	Comment^*b*^
Amphotericin B	2		
C. glabrata ML72254		>32	R
C. glabrata ATCC 90030		0.5	S
Fluconazole	64		
C. glabrata ML72254		>256	R
C. glabrata ATCC 90030		4	S
Voriconazole	0.03		
C. glabrata ML72254		>32	R
C. glabrata ATCC 90030		0.03	S
Anidulafungin	0.03		
C. glabrata ML72254		0.015	S
C. glabrata ATCC 90030		0.015	S
Micafungin	0.03		
C. glabrata ML72254		0.008	S
C. glabrata ATCC 90030		0.015	S

a ECOFF: Epidemiological cutoff value (47, 48).

b S, susceptible; R, resistant.

### WGS data analysis summary.

Approximately 37,000 variants were detected in the coding regions of the ML72254 genome. Of those, 70% were synonymous variants. The detected nonsynonymous mutations in the genes involved in late-pathway ergosterol synthesis, sterol uptake, and *PDR1* gene mutations are summarized in Table 2. The matching multilocus sequence type (MLST) profile was identified as sequence type 7 (ST7) for ML72254.

**TABLE 2:** Some nonsynonymous variations detected in the genome of the Candida glabrata ML72254 strain

Gene	Locus tag	Description	Nucleotide change	Amino acid change
Mutations of the genes involved in late-pathway ergosterol synthesis				
*ERG1*	CAGL0D05940g	Squalene monooxygenase	941delC	P314fs
*ERG2*	CAGL0L10714g	C-8 sterol isomerase	619A→G	I207V
*ERG4*	CAGL0A00429g	Delta(24(24(1)))-sterol reductase	38C→A	T13N
*ERG6*	CAGL0H04653g	Sterol 24-C-methyltransferase	143G→A	R48K
*ERG7*	CAGL0J10824g	Lanosterol synthase	2194A→G	T732A
*ERG8*	CAGL0F03993g	Phosphomevalonate kinase	1343A→G	N448S
*ERG10*	CAGL0L12364g	Acetyl-CoA C-acetyltransferase	508A→G	N170D
*ERG24*	CAGL0I02970g	Delta14-sterol reductase	526G→C	V176L
Mutations of the genes involved in sterol uptake				
*AUS1*	CAGL0F01419g	ATP-binding cassette transporter	4139T→A	F1380Y
			103C→T	H35Y
*PDR1* gene mutations				
*PDR1*	CAGL0M13827g	Zinc finger transcription factor	226T→C	S76P
			271G→A	V91I
			293T→C	L98S
			427A→C	T143P

### Deletion mutation in the *ERG1* gene.

**FIG 4:**
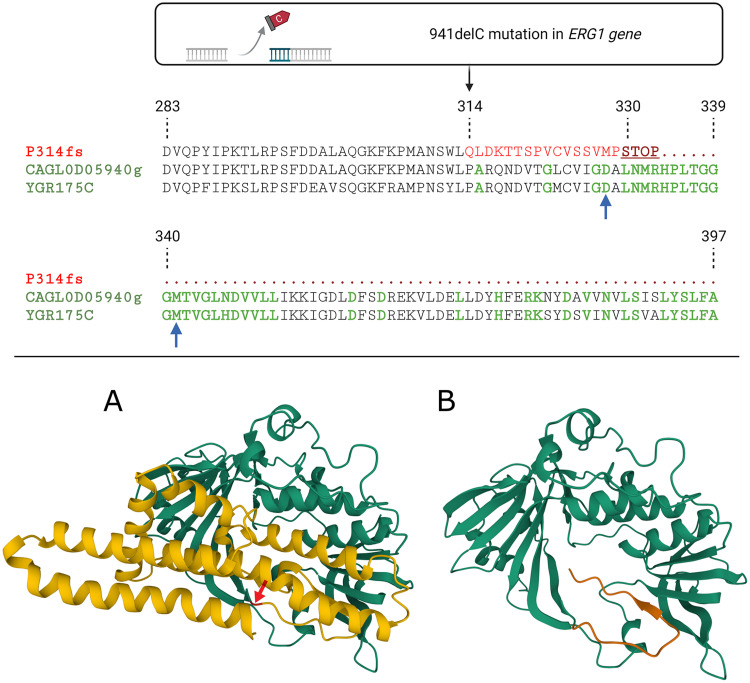
The upper panel shows a multiple sequence alignment of frameshift mutant *ERG1* (P314fs), Candida glabrata wild-type *ERG1* (CAGL0D05940g), and Saccharomyces cerevisiae *ERG1* (YGR175C) translated sequences between positions 283 and 397. A cytosine deletion at position 941 in the *ERG1* gene leads to a frameshift mutation (Pro314fs). This mutation initially created a stop codon at position 330 in the translated sequence, thereby shortening the primary sequence of squalene monooxygenase by 159 amino acids at the C terminus. This mutation affected many conserved amino acid patterns (shown in green) and two essential FAD-binding amino acids (indicated with blue arrows). (A and B) The AlphaFold Colab predicted structures of wild-type C. glabrata Erg1p (A) and P314fs mutant Erg1p (B) displayed visual differences. These included a proline residue at position 314 (indicated with a red arrow) and amino acid residues affected by frameshift mutation (colored yellow) in the wild-type C. glabrata Erg1p structure (A) and amino acid residues in positions 314 to 329 (colored orange) in the P314fs mutant Erg1p structure (B).

A novel cytosine nucleotide deletion was detected at position 941 (941delC) in the *ERG1* gene of the ML72254 genome. The *ERG1* gene (CAGL0D05940g) codes a 489-amino-acid-long squalene monooxygenase enzyme (Erg1p), which is located in the endoplasmic reticulum (ER) membrane. This enzyme catalyzes a stereospecific epoxidation of squalene to (S)-2,3-epoxysqualene (26). This squalene oxidation is the second reaction of late-pathway ergosterol synthesis (Fig. 1). A UniProt search revealed multiple flavin adenine dinucleotide (FAD)-binding sites in the C. glabrata Erg1p, similar to that of the Saccharomyces cerevisiae Erg1p (YGR175C). The 941delC mutation in the *ERG1* gene caused a frameshift at Pro314 (P314fs) and formed a stop codon at position 330 in the *ERG1* translated sequence, ultimately leading to synthesis of a truncated protein. The mutation eliminated some FAD-binding sites and many conserved amino acid patterns (Fig. 4). The squalene epoxidase domain (pfam08491), which spans positions 194 to 466, was damaged by the P314fs mutation. A visual comparison of the wild-type Erg1p predicted structure and the P314fs Erg1p predicted structure revealed vast differences (Fig. 4). These findings indicated that the 941delC mutation severely damaged the protein function.

### The predictive ability of the computational biology workflow.

The compatibility of the predicted outcomes and previous experimental data are detailed in Table 3. For two variations (R48K in Erg6p and I207V in Erg2), Missense3D detected no structural damage, and the PROVEAN classification was neutral. Although three variations (T121V and T121I in Erg2 and V126F in Erg6p) were previously shown to disrupt the protein function, Missense3D did not detect a structural defect (27, 28). PROVEAN analysis classified these three mutations as deleterious. For the remaining four variations (G119S and G122S in Erg2p, C198F in Erg6p, and G315D in Erg11p), whose effects on the phenotype were proven previously, Missense3D gave a “structural damage detected” warning, while PROVEAN analysis classified the variations as deleterious (27–30).

**TABLE 3:** Comparison of Missense3D and PROVEAN predictions with known effects of previously described missense mutations, including detailed Missense3D structural analysis results and PROVEAN scores

Gene	Variation	Features and experimentally shown effects of variations	Missense3D structural damage analysis^*a*^	PROVEAN result
*ERG2*	G119S	This amino acid substitution is located in proximity to the enzyme active site (sterol Δ8-Δ7 isomerization). This amino acid substitution impairs Erg2p function (27).	Structural damage detected This substitution triggers a disallowed phi/psi alert. The phi/psi angles are in a favored region for the wild-type residue but in an outlier region for the mutant residue.	Score: −6; deleterious
*ERG2*	G122S	This amino acid substitution is located in proximity to the enzyme active site (sterol Δ8-Δ7 isomerization). This amino acid substitution impairs Erg2p function (27).	Structural damage detected This substitution triggers a disallowed phi/psi alert. The phi/psi angles are in a favored region for the wild-type residue but in an outlier region for the mutant residue. This substitution replaces a buried GLY residue (RSA, 0.0%) with a buried SER residue (RSA, 3.0%).	Score: −6; deleterious
*ERG2*	I207V	This amino acid substitution does not impair protein function (27, 28).	No structural damage detected	Score: 0.785; neutral
*ERG2*	T121V	The corresponding amino acid in Saccharomyces cerevisiae is involved in sterol Δ8-Δ7 isomerization. This amino acid substitution impairs Erg2p function (28).	No structural damage detected	Score: −4.094; deleterious
*ERG2*	T121I	The corresponding amino acid in Saccharomyces cerevisiae is involved in sterol Δ8-Δ7 isomerization. This amino acid substitution impairs Erg2p function (28).	No structural damage detected	Score: −4.774; deleterious
*ERG6*	R48K	Does not effect protein function (27)	No structural damage detected	Score: 1.502; neutral
*ERG6*	V126F	This amino acid substitution is located in a conserved amino acid sequence pattern. This amino acid substitution impairs Erg6p function (27).	No structural damage detected	Score: −4.644; deleterious
*ERG6*	C198F	This amino acid substitution is located in a conserved amino acid sequence pattern. This amino acid substitution impairs Erg6p function (29).	Structural damage detected This substitution disrupts all side-chain/side-chain H-bond(s) and/or side-chain/main-chain bond(s) H-bonds formed by a buried CYS residue (RSA 2.2%).	Score: −3.783; deleterious
*ERG11*	G315D	This amino acid substitution is located in a CYP51-like conserved domain heme-binding site. This amino acid substitution impairs Erg11p function (30).	Structural damage detected The substitution leads to the contraction of cavity vol by 79.272 Å^3^.	Score: −5.485; deleterious

a GLY, glycine; RSA, relative solvent accessibility; SER, serine.

### Evaluation of the detected missense variations of ML72274 in genes responsible for ergosterol synthesis using computational biology workflow.

Seven amino acid substitutions were detected throughout the ergosterol synthesis pathway of ML72254. Five of these missense variations (R48K in Erg6p, I207V in Erg2p, T13N in Erg4p, T732A in Erg7p, and V176L in Erg24p) were evaluated using computational biology workflow. Neither PROVEAN nor Missense3D indicated any possible deleterious impact on protein function regarding these amino acid substitutions, as summarized in Table 4. None of these amino acid substitutions were located in the conserved sequence patterns. The R48K and I207V variations were examined in previous studies and were used here to test the performance of computational biology applications in this study (27, 28). No further laboratory experiments were conducted for these five variations, which were initially considered to represent natural amino acid polymorphisms.

**TABLE 4:** Evaluation of the detected missense variations in the ergosterol synthesis proteins of the ML72254 strain using Missense3D and PROVEAN

Protein	Amino acid substitution	Features of the amino acid substitution	Missense3D result	PROVEAN result
Erg6p	R48K	Protein sequence alignment of Erg6p in CGD revealed that Candida albicans, C. parapsilosis, C. dubliniensis, and C. tropicalis have lysine (K) instead of arginine (R) at the corresponding position.	No structural damage detected	Score: 1.502; neutral
Erg2p	I207V	Protein sequence alignment of Erg2p in CGD revealed that Saccharomyces cerevisiae and Candida auris homologous sequences are occupied by valine (V) instead of isoleucine (I) at the corresponding position.	No structural damage detected	Score: 0.785; neutral
Erg4p	T13N	This substitution replaced a neutral polar amino acid with another neutral polar amino acid. The amino acid substitution was located in a variable region in different yeast species.	No structural damage detected	Score: −0.3; neutral
Erg7p	T732A	This substitution was located next to the C-terminal amino acid residue of Erg7p, a variable region in different yeast species.	No structural damage detected	Score: 0.3; neutral
Erg24p	V176L	This substitution replaced a hydrophobic amino acid with another hydrophobic amino acid. Protein sequence alignment of Erg24p in CGD revealed that Candida auris and C. tropicalis have leucine (L) instead of valine (V) at the corresponding position.	No structural damage detected	Score: −0.5; neutral

The remaining two amino acid variations were N448S in Erg8p (CAGL0F03993g) and N170D in Erg10p (CAGL0L12364g). Because C. glabrata laboratory strain BG2 carries a serine amino acid at position 448 in Erg8p and aspartate at position 170 in Erg10p, as indicated by a blastp search, these two variations were interpreted as naturally occurring amino acid polymorphisms.

No nonsynonymous mutations involved in late-pathway ergosterol synthesis were detected in *ERG3* (CAGL0F01793g), *ERG5* (CAGL0M07656g), *ERG9* (CAGL0M07095g), or *ERG11* (CAGL0E04334g). Only synonymous mutations were detected in other genes involved in the mevalonate pathway (Fig. 1).

### Variations in genes involved in sterol uptake.

F1380Y and H35Y amino acid substitutions were detected in the Aus1p (CAGL0F01419g). PROVEAN classified these mutations as neutral. Only synonymous mutations were detected in the *UPC2B* and *TIR3* sequences.

### Variations in other genes.

The *PDR1* gene regulates ABC transporter genes, and gain of function mutations of the *PDR1* gene cause azole resistance (31). S76P, V91I, L98S, and T143P amino acid substitutions were detected in the *PDR1* gene translated sequence. Only synonymous mutations were detected in *FKS1* and *FKS2* genes, which have hot spot regions related to echinocandin resistance (32, 33). A well-known *MSH2* (which codes a protein involved in DNA mismatch repair) point mutation, V239L, was also detected in the ML72254 isolate (34).

## DISCUSSION

In this study, a cholesterol-dependent C. glabrata clinical isolate that confers azole and AmB resistance was investigated using artificial intelligence (AI) technologies and cloud computing applications. This is the first of the known cholesterol-dependent C. glabrata isolates to be found in Turkey. A novel mutation in the *ERG1* gene, 941delC, was identified as the sole causative molecular change for this phenotype, since all the other amino acid substitutions in ergosterol synthetic proteins had no effect on the protein functions, as predicted by web-based computational biology applications. Although the effects of the missense mutations in ML72254 have not been experimentally demonstrated, the predictions of the computational biology workflow were comparable to previous experimental results, as shown by this study.

Cholesterol-dependent C. glabrata isolates have been previously recovered from different samples (e.g., urine, blood cultures, nasopharyngeal swabs) (23, 24). As seen for the ML72254 strain, those previously isolated clinical strains failed to grow on SDA alone but grew on SDA following the addition of human serum or sheep blood. Subsequent sterol analyses showed that cholesterol-dependent C. glabrata clinical isolates had no activities of the squalene monooxygenase encoded by *ERG1* or the lanosterol synthase encoded by *ERG7* (27). Because the defects in Erg1p and Erg7p occurred before the first sterol molecule (lanosterol) was synthesized, those isolates became totally dependent on imported sterols (35). Similar to our findings, supplementation of cholesterol (60 μg/mL) supported robust growth for those isolates (35). Quantitative measuring of sterol content of ML72254 was not performed in the present study. However, total dependence on imported sterols under aerobic conditions and the position of the *ERG1* gene mutation points out that the ML72254 strain could not produce ergosterol at all.

Cholesterol-dependent isolates use imported cholesterol as a membrane sterol. Since AmB preferentially binds to ergosterol over cholesterol, the cholesterol-dependent variants are expected to gain AmB resistance, as seen in the ML72254 strain. There are many known mechanisms that cause azole resistance to C. glabrata. However, common resistance mechanisms were not found in the strain. Mutations in the ML72254 *PRD1* gene (S76P, V91I, L98S, and T143P) can also be seen in azole-susceptible C. glabrata isolates, indicating no relationship to azole resistance as shown in the past studies (36, 37). Because of this, azole resistance of the ML72254 strain could not be explained by the upregulation of membrane transporters which were described previously (31). Azole resistance was not linked to the modifications of the target molecule, lanosterol 14-alpha-demethylase, because only synonymous variations were detected in *ERG11*. The cholesterol-dependent nature of the ML72254 strain was solely responsible for azole resistance. Cholesterol uptake causes azole resistance in cholesterol-dependent strains as shown previously (21). As expected from the *FKS1* and *FKS2* sequences, the ML72254 isolate was susceptible to echinocandin antifungals.

Two cholesterol-dependent C. glabrata isolates, Kw1018/12 and Kw1154/12, reported from Kuwait, were resistant to AmB (MIC, ≥32 μg/mL) and azoles (fluconazole MIC, ≥256). The *ERG11* and *ERG3* gene sequences of Kw1018/12 and Kw1154/12 showed only synonymous mutations within the coding regions. The reported MIC values were identical to our findings; however, *ERG1* gene sequencing was not performed in that study (38). It would be very exciting to know whether these two isolates have mutations in *ERG1* genes or not.

Interestingly, in another study, the C. glabrata *ERG1* mutant (CgTn201S), which shows increased fluconazole susceptibility and a slightly elevated MIC for AmB compared to the original strain (Cg1660), was identified by transposon mutagenesis. Interruption of the *ERG1* open reading frame (ORF) at codon 476 (13 amino acids away from the C terminus) decreased the level of ergosterol synthesis in CgTn201S but did not eliminate it. Although the uptake of exogenous cholesterol was enhanced in CgTn201S, it could grow aerobically without cholesterol supplementation (39). In contrast, a frameshift occurring 175 amino acids from the C terminus in *ERG1* ORF of ML72254 can elucidate the differences in growth characteristics observed between ML72254 and CgTn201S. The fluconazole (FL) susceptibility of CgTn201S was reduced by the exogenous addition of cholesterol. Under hypoxic conditions, which further suppressed the mutant Erg1p function in CgTn201S, this effect became more dramatic (FL MIC, ≥256 μg/mL) (39). However, under aerobic conditions, ML72254 showed azole resistance (FL MIC, ≥256 μg/mL).

A different study has shown upregulation of *AUS1*, *UPC2B*, and *TIR3* expression in CgTn201S compared to that of wild-type cells (40), and these genes were responsible for sterol uptake in CgTn201S. In the present study, no mutation was interpreted as possibly deleterious regarding the *AUS1* gene of ML72254, and no amino acid substitution was detected in Upc2bp or Tir3p. These findings were anticipated since ML72254 was viable only in sterol-rich environments.

Another clinical C. glabrata isolate (CG156) harboring a missense mutation (G315D) in *ERG11* and showing fluconazole (MIC, ≥256 μg/mL) and AmB (MIC, 16 μg/mL) resistance was identified previously (30). The researchers reported that CG156 could grow on sterol-free minimal medium despite its lack of Erg11p activity. Other studies have shown that a C198F amino acid substitution and a nonsense mutation at codon Gln332 in *ERG6* were associated with reduced susceptibility to AmB and increased susceptibility to azole antifungals (29, 41). The findings of the present study and all the studies mentioned above confirm that mutations in the ergosterol synthesis pathway cause a spectrum of phenotypic changes in C. glabrata. Interrupting the early steps of late-pathway ergosterol synthesis reduces cellular viability severely under standard growth conditions.

Another interesting finding was the pseudohypha-like growth of ML72254 on the SDA-HS medium. C. glabrata does not produce hyphae or pseudohyphae except under special culture conditions such as CO_2_ exposure or nitrogen starvation (42, 43). Similar microscopic features have been observed for C. glabrata isolates with mutations in *ERG11* or *ERG6* (29, 30). This behavior of ML72254 could be related to defective ergosterol synthesis. Since pseudohypha-like formations were observed explicitly for the cells grown on the SDA-HS medium, it is hard to exclude the effect of medium composition. Further research should be undertaken to investigate genomic changes leading to pseudohypha-like formation.

Unlike previous studies, WGS was employed in the present study. WGS allowed us to inspect a broader range of genes. This study presented a different concept—the use of web-based bioinformatic tools—to sort out missense variations in the C. glabrata ergosterol synthesis pathway. PROVEAN and Missense3D were used concurrently to minimize potential false-negative predictions of variant effects. A comprehensive evaluation of this workflow was not possible since few of these variations were reported in the literature and due to the lack of Protein Data Bank (PDB) structures. Nevertheless, this workflow can be helpful when assessing large amounts of missense variation data before further laboratory experiments.

The PROVEAN results were perfectly aligned with previous sterol analysis results, as presented in our study. However, the Missense3D analysis resulted in false negatives for three mutations previously known to affect protein function. Some false-negative results are anticipated because the accuracy of Missense3D depends on the structure model used. Another vital point is that Missense3D analysis does not take into account some features, such as residue critical function or ligand binding. Missense3D is applicable to protein structures obtained by experimental results and by homology modeling, as indicated by previous literature (44). Because the homology modeling results for ergosterol synthesis proteins of C. glabrata yielded low-quality and low-sequence coverage, the AI-predicted structures were used in the Missense3D analysis step in our study. Recently, the Missense3D platform announced that AlphaFold-predicted structures could be used for analysis. To our knowledge, this is the first use of protein structures predicted by AlphaFold Colab to evaluate possible biochemical changes introduced by missense mutations in a clinical fungal isolate.

The Missense3D results obtained using AI-predicted structures may provide valuable information about drug resistance mechanisms in C. glabrata. As shown by the present research, AlphaFold (or the Colab version) is a promising tool for discovering medically important fungal protein structures. All the applications chosen for this study were open-access and easy to use.

In summary, cholesterol-dependent C. glabrata isolates can cause persisting candidemia, as seen in our case, because of their slow-growing nature and multidrug resistance. Bactec FX managed to detect the growth of these isolates despite their slow-growing nature. Identification with MALDI-TOF was reliable for ML72254, despite its mutation in the *ERG1* gene. No definitive study has yet described the incidence of cholesterol-dependent C. glabrata clinical isolates, and because cholesterol-dependent C. glabrata isolates are rarely isolated in clinical samples, they can easily be overlooked during routine laboratory procedures. Microbiologists, therefore, need to be alert when discrepancies occur between microscopic examination and growth on routine media. In addition, because these isolates confer antifungal resistance, patient management requires extra care. Our study defined a new genetic change in the *ERG1* gene, 941delC, which blocks ergosterol synthesis in C. glabrata and confers a cholesterol dependency and azole and AmB resistance. Our findings also confirmed that web-based computational biology solutions, such as PROVEAN, AlphaFold Colab, and Missense3D, can be used to examine the possible impacts of missense mutations in C. glabrata.

## MATERIALS AND METHODS

### Isolation of ML72254.

Eleven yeast isolates with similar growth characteristics had been recovered from consecutive blood cultures (Bactec-FX; Becton, Dickinson, Sparks, MD, USA) of a 63-year-old male patient with COVID-19. He was also being treated for inflammatory bowel disease-related gastrointestinal hemorrhagia in the intensive care unit. Gram staining performed from blood culture bottles gave positive signals. When yeast cells were detected, the blood culture bottles were subcultured into Columbia agar with 5% sheep blood (SBA; BD, New Jersey, USA) and into SDA (BBL SDA; Becton, Dickinson) and a chromogenic medium (Baltimore Biological Laboratory (BBL) CHROMagar *Candida* medium; BD GmbH, Germany). After a 72-h incubation, small colonies were visible only on the SBA plates and not on the other media. One of the patient’s isolates, ML72254, was chosen for further processing.

### Species identification.

Biochemical identification with API ID 32 C (bioMérieux, Marcy-l’Étoile, France) could not be made because the isolate did not reproduce in this system. The ML72254 strain was therefore incubated for 120 h on an SBA plate, followed by protein extraction with formic acid and acetonitrile, and matrix-assisted laser desorption ionization–time of flight (MALDI-TOF; Bruker Microflex LT system; Bruker Daltonics, US) was used for species identification. A threshold score of ≥2 was set as the criterion for accurate species-level identification.

### Observations of growth characteristics.

The ML72254 strain and a C. glabrata control strain (ATCC 90030) were incubated for 72 h at 37°C in SBA medium, and then a loopful of each culture was suspended in physiological saline. Each suspension was diluted to give concentrations of 2 × 10^7^, 2 × 10^6^, 2 × 10^5^, and 2 × 10^4^ cells/mL, determined by hemocytometer counts under a light microscope according to the recommendations (39). Then, 10 μL of each concentration was then added to solid media (SBA plates, SDA plates supplemented with human serum [SDA-HS plates], and SDA plates supplemented with 60 μg/mL cholesterol [SDA-CS plates]) to give a series of spots containing 2 × 10^5^ to 2 × 10^2^ cells (35, 39). These cultures were incubated in ambient air at 37°C for 120 h. Colony sizes and morphologies were observed every 24 h until adequate growth was achieved. The microscopic features of cells grown on SDA-HS were also examined.

### Antifungal susceptibility testing.

Use of the broth microdilution method (CLSI M27) was not possible for antifungal susceptibility testing of ML72254, as no growth was detected in the RPMI 1640 medium. The susceptibility to different antifungal drugs (amphotericin B, fluconazole, voriconazole, anidulafungin, and micafungin) was determined in cultures grown on Mueller-Hinton agar supplemented with 5% sheep blood using a modified Etest (bioMérieux, Marcy l’Étoile, France) gradient diffusion method (45, 46). C. parapsilosis ATCC 22019 and C. krusei ATCC 6258 were used for quality control, while C. glabrata ATCC 90030 was used as an example of a susceptible C. glabrata strain. The Etest MIC values were read after 48 to 120 h, depending on the growth of the strain, and were interpreted as susceptible (S)/resistant (R) according to the recently published Etest epidemiological cutoff values (47, 48).

### Whole-genome sequencing.

Whole-genome sequencing (WGS) was performed to confirm the species identification and to determine the genomic variations of the ML72254 strain. DNA was extracted from cells after a 120-h incubation on SBA plates using the Quick-DNA fungal/bacterial kit (Zymo Research, California, USA) and following the manufacturer’s recommendations. A library was prepared using the Nextera DNA library prep kit (Illumina, California, USA). The Illumina NextSeq 550 next-generation sequencing platform was used for the sequencing study.

### *In silico* analysis of WGS data and variant detection.

The WGS data were analyzed using Galaxy (https://usegalaxy.eu/), an open-source web-based platform for computational biology applications (49). Quality control of the raw reads was done using FastQC (Galaxy version 0.73). Adapter sequences and low-quality reads were filtered using Trimmomatic (Galaxy version 0.38.1) (50). The filtered reads were alligned to the reference C. glabrata strain CBS138 genome (ASM254v2) with Bowtie2 (Galaxy version 2.4.2) (51). Variants were called with Lofreq (Galaxy version 2.1.5) and filtered based on the criteria of variant call quality (minimum = 20), proportion of reads supporting the variant (>0.9), and strand bias (52). Variants in coding regions were selected and annotated using SnpEff (Galaxy version 4.3) (53). Variants were explored for single nucleotide variations (SNVs) and indels in genes related to ergosterol synthesis and antifungal drug resistance. The genome was assembled using SPAdes (Galaxy version 3.12.0) (54). The assembly was then uploaded to the PubMLST portal (https://pubmlst.org/) to identify the sequence type (55, 56).

### Sorting amino acid substitutions using web-based computational biology platforms.

**FIG 5:**
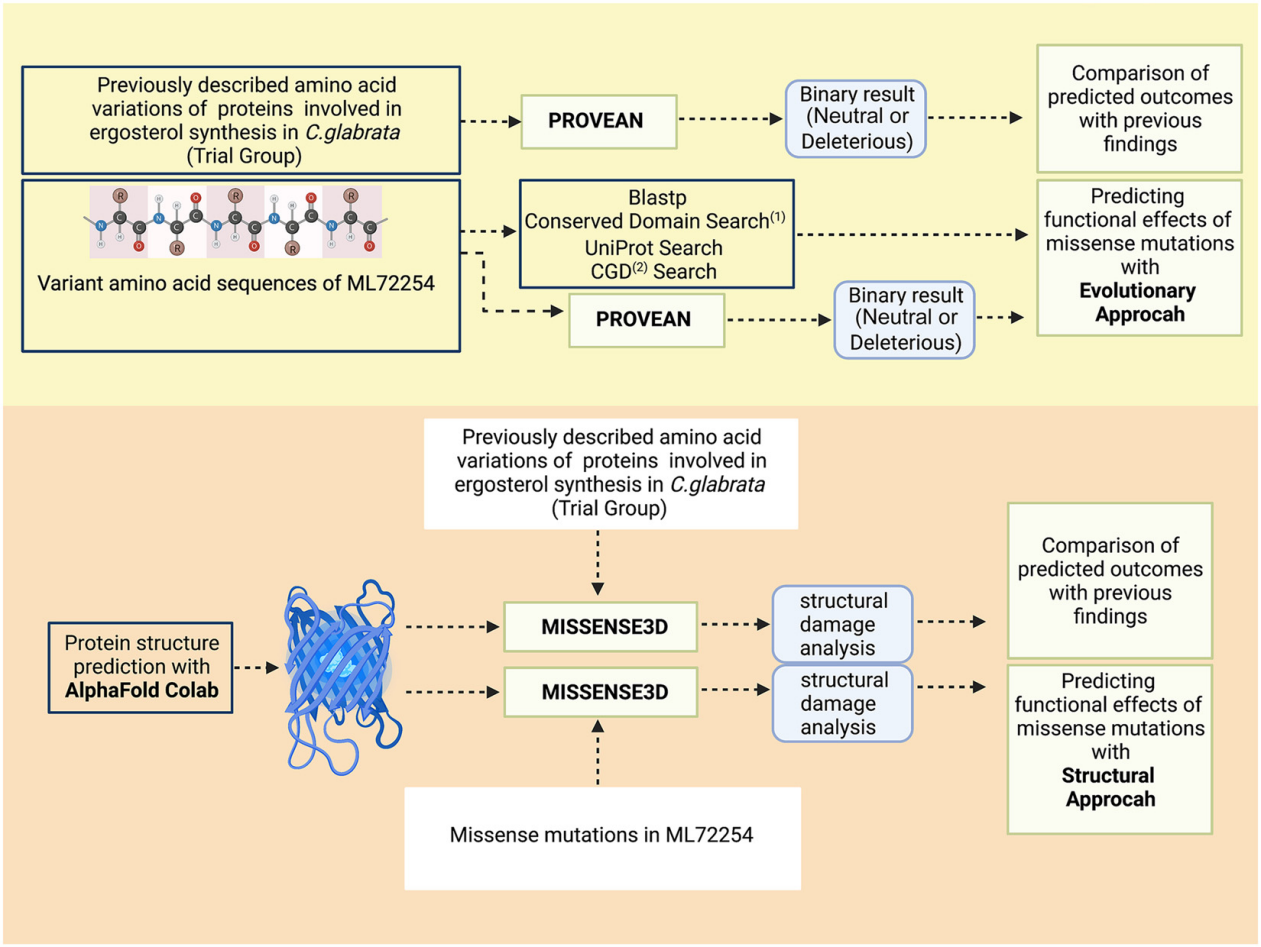
Computational biology workflow. The upper panel summarizes the evolutionary approach. The lower panel summarizes the structural approach. The PROVEAN and Missense3D applications were tested using previously described amino acid variations (trial group) related to ergosterol synthesis in Candida glabrata. (1) NCBI Conserved Domain search after performing blastp operation. (2) CGD, *Candida* Genome Database.

In addition to a cytosine deletion in the *ERG1* gene, many missense mutations were detected during the variant analysis step in the genes associated with the late pathway of ergosterol synthesis and its regulation. Designing laboratory experiments to test how each amino acid substitution affected ergosterol synthesis and antifungal susceptibility would require a substantial amount of resources; consequently, a computational biology workflow that utilizes web-based computational biology applications was constructed to sort these amino acid substitutions in this study (summarized in Fig. 5). This allowed accurate predictions of the possible impacts of these mutations on phenotypic characteristics.

Two different approaches—evolutionary and structural—were adopted to create this workflow. The evolutionary approach was based on the idea that mutations in the conserved sequence patterns among species are more likely to have a phenotypic effect. Here, variant amino acid sequences were subjected to a BLAST search against protein sequence databases to find similar sequence patterns that were submitted previously. Positions of the mutations were inspected on the NCBI Conserved Domains webpage (https://www.ncbi.nlm.nih.gov/Structure/cdd/wrpsb.cgi). Homolog amino acid sequences of closely related species were also examined on the *Candida* Genome Database (CGD) (http://www.candidagenome.org/) (57). The PROVEAN Protein (http://provean.jcvi.org/seq_submit.php) web application was used to calculate the functional effect of sequence variations, as previously described (58). This application generates a PROVEAN score and conducts binary classifications of amino acid substitutions. If the PROVEAN score is less than the −2.5 threshold value, the tool classifies the mutation as “deleterious,” indicating a high probability of functional damage. Otherwise, it ranks the mutation as “neutral.”

The idea behind the structural approach was that a missense mutation that disrupts the stable protein structure, which is the most conserved biological information, would be expected to affect the function of that protein. In this step, predicted structure models for key proteins were created using AlphaFold Colab Notebook (AlphaFold.ipynb), a cloud computing service. AlphaFold is an artificial intelligence (AI) program developed by DeepMind and makes accurate protein structure predictions starting from amino acid sequences (59, 60). AlphaFold Colab Notebook uses a slightly simplified version of AlphaFold 2. Running AlphaFold locally demands programming skills and substantial computing power. For that reason, the AlphaFold Colab Notebook version has become a fast, easy-to-use, and cost-efficient alternative, with only tiny drops in model accuracy compared to the local version. After model prediction with AlphaFold Colab, the structure files were uploaded to another web-based application, Missense3D (http://missense3d.bc.ic.ac.uk/~missense3d/). Missense3D predicts significant structural changes introduced by amino acid substitutions (44). If Missense3D detects at least 1 of the 16 structural alterations it looks for, it gives a “structural damage detected” warning and provides qualitative descriptions of potentially damaging variants.

Finally, previously known amino acid substitutions of C. glabrata (G119S, G122S, I207V, T121V, and T121I in Erg2p; R48K, V126F, and C198F in Erg6p; and G315D in Erg11p) were used to create a trial group to test the usability of these two web-based applications (PROVEAN and Missense3D) in our scenario (27–30). Two of the nine variations—I207V in Erg2p and R48K in Erg6p—had not previously been associated with a deleterious functional effect on proteins (27, 28), while other previous experiments using sterol analysis methods had confirmed that the other seven mutations were related to impaired ergosterol synthesis. We examined the predictive ability of the described computational biology workflow to evaluate the missense mutations detected in the ML77254 genome.

### Data availability.

The raw sequence data have been deposited at the Sequence Read Archive under BioProject accession number PRJNA786916. This WGS project has been deposited at DDBJ/ENA/GenBank under the accession number JAKLYX000000000. The version described in this paper is version JAKLYX010000000.
